# Comparison of spectroscopy technologies for improved monitoring of cell culture processes in miniature bioreactors

**DOI:** 10.1002/btpr.2459

**Published:** 2017-03-29

**Authors:** Ruth C. Rowland‐Jones, Frans van den Berg, Andrew J. Racher, Elaine B. Martin, Colin Jaques

**Affiliations:** ^1^ BBTC, Newcastle UniversityNewcastle Upon TyneNE1 7RUU.K.; ^2^ Lonza Biologics plc228 Bath RoadSloughSL1 4DXU.K.; ^3^ University of CopenhagenRolighedsvej 30FrederiksbergDK‐1958Denmark; ^4^ School of Chemical and Process EngineeringUniversity of LeedsLeedsLS2 9JTU.K.

**Keywords:** Raman spectroscopy, near‐infrared spectroscopy, 2D‐fluorescence, process analytical technology (PAT), design of experiments

## Abstract

Cell culture process development requires the screening of large numbers of cell lines and process conditions. The development of miniature bioreactor systems has increased the throughput of such studies; however, there are limitations with their use. One important constraint is the limited number of offline samples that can be taken compared to those taken for monitoring cultures in large‐scale bioreactors. The small volume of miniature bioreactor cultures (15 mL) is incompatible with the large sample volume (600 µL) required for bioanalysers routinely used. Spectroscopy technologies may be used to resolve this limitation. The purpose of this study was to compare the use of NIR, Raman, and 2D‐fluorescence to measure multiple analytes simultaneously in volumes suitable for daily monitoring of a miniature bioreactor system. A novel design‐of‐experiment approach is described that utilizes previously analyzed cell culture supernatant to assess metabolite concentrations under various conditions while providing optimal coverage of the desired design space. Multivariate data analysis techniques were used to develop predictive models. Model performance was compared to determine which technology is more suitable for this application. 2D‐fluorescence could more accurately measure ammonium concentration (RMSE_CV_ 0.031 g L^−1^) than Raman and NIR. Raman spectroscopy, however, was more robust at measuring lactate and glucose concentrations (RMSE_CV_ 1.11 and 0.92 g L^−1^, respectively) than the other two techniques. The findings suggest that Raman spectroscopy is more suited for this application than NIR and 2D‐fluorescence. The implementation of Raman spectroscopy increases at‐line measuring capabilities, enabling daily monitoring of key cell culture components within miniature bioreactor cultures. © 2017 American Institute of Chemical Engineers *Biotechnol. Prog.*, 33:337–346, 2017

## Introduction

The industry in recent years has become increasingly interested in the use of spectroscopy technologies such as Raman, near‐infrared (NIR), and 2D‐fluorescence (2D‐F) for the measuring, monitoring, and control of bioprocesses. This is, in part, a result of the American Food and Drug Administration's (FDA) guidance on process analytical technology (PAT), which encourages the use of innovative tools and technologies to increase understanding and control of manufacturing processes.^1^ Since the publication of this work, there has been an increasing move to develop biopharmaceutical processes using a Quality by Design (QbD) approach. The ICH Q8 guideline defines QbD as “a systematic approach to development that begins with predefined objectives and emphasizes product and process understanding and process control, based on sound science and quality risk management.”^2^ Spectroscopy technologies offer a nondestructive, rapid, and robust method for generating multianalyte data.^3^ These technologies are used routinely to monitor chemical pharmaceutical production processes, but their application to mammalian cell culture for production of biologics is much more challenging. This is primarily due to the complex matrix background^4^ and low concentrations of the metabolites of interest. Despite these challenges, there is a growing body of literature demonstrating their ability to measure multiple analytes simultaneously, such as glucose, ammonium, and lactate in mammalian cell culture.^5^, ^6^, ^7^, ^8^, ^9^, ^10^ The primary focus, however, has been on their use for online monitoring at laboratory scale (e.g., 5–10 L). The applicability of spectroscopic methods for miniature bioreactor (MB) cultures has been reviewed^11^; however, the authors are not aware of any literature experimentally comparing the ability of spectroscopy techniques to monitor multiple analytes under suitable operational constraints required for daily monitoring of MB cultures.

MB are widely utilized in the development of biologics. Cell culture process development requires the screening of large numbers of cell lines. Traditionally, this has been carried out using shaken flask cultures with successful cell lines progressing to bench‐top bioreactors and on to pilot‐scale studies.^12^ Shaken flask cultures are used due to their ease of operation with few mechanical complications^12^ coupled with low costs. There are, however, limitations with shaken flask cultures, including gas transfer and continuous monitoring.^13^ In response to these limitations, considerable effort has been invested in the development of MB systems that can better model the physiochemical environment experienced by cells in laboratory‐scale bioreactor cultures.

The ambr™ system (Sartorius Stedim) is a commercially available MB system that mimics many of the characteristics of laboratory‐scale bioreactors. Each vessel has an internal impeller, closed loop control for pH and dO_2_ and independent control of O_2_ and CO_2_ gas flow rates. Several studies have been carried out demonstrating that culture performance in laboratory‐scale, stirred‐tank bioreactors is more comparable to that in MBs than in traditional shaken flask cultures^14^, ^15^ and that process and product characteristics of MBs are comparable to laboratory‐scale.^16^ There are still, however, a number of limitations including lack of geometric similarity with large‐scale vessels^12^ and desirable operational controls, for example, continuous feeding.^15^ pH, temperature, and dO_2_ are measured online for the MB system. In current laboratory‐scale bioreactors, however, additional analytes are often measured to improve control of the nutritional environment and facilitate process improvement. For example, glucose, lactate, and ammonium are routinely measured by taking offline measurements with bioanalysers such as Nova BioProfile 400 (Nova Biomedical). The small volume of MB cultures (15 mL) is, however, incompatible with the large sample volume required for this analyser (600 µL), preventing daily offline samples.^11^ Moreover, it would take approximately 3 h to analyze all 48 vessels of a typical MB system. This constraint results in a tradeoff between data quantity and experimental throughput,^12^ and spectroscopy technologies may be used to resolve this constraint. This study compares the ability of NIR, Raman, and 2D‐F for measuring analytes in cell culture supernatant under constraints imposed by MB cultures to allow daily monitoring:

1. Analytes must be measured for all 48 MB vessels within 1 h
2. Sample volume must be <50 µL

These constraints are specific to MB systems. Large‐scale bioreactors often implement a continuous feeding strategy for glucose. This cannot be performed for current MB systems. To mimic the feeding strategy at larger scale, glucose is fed every 8 h. A control feedback loop is used to alter the glucose feed rate as a function of the glucose concentration and the volume of cell culture currently within the vessel. To minimize the time between taking an offline measurement and feeding glucose, all results must be obtained within 1 h. As such, the total acquisition times were minimized to meet the time constraint required of the current feeding strategy. The working volume of each vessel is 10–15 mL. Excessive removal of the culture throughout the process will lower the working volume below this range, changing the culture conditions. This can lead to detrimental effects on culture performance as lower volumes will increase oxygen uptake causing aberrant foaming and lack of DO control. As a result, culture removal needs to be minimized. Small sample sizes of <50 µL must therefore be used to reduce the amount of culture being removed from each vessel to allow daily offline monitoring of MB cultures.

A design‐of‐experiment (DOE) approach was used to select from a library of cell culture supernatant samples. Spectra were acquired by the 3 spectroscopy technologies for each sample under the operational constraints required for daily monitoring of a MB system. A range of multivariate data analysis (MVDA) and data handling techniques were investigated to develop predictive models for each analyte, including partial least squares (PLS), unfolded‐PLS,^17^ multiway PLS (NPLS^18^), and parallel factor analysis (PARAFAC). The performance of each model was compared to determine which spectroscopy technology would be more suitable for measuring the 3 analytes.

## Materials and Methods

### Experimental design

Three analytes were considered in this investigation: glucose, lactate, and ammonium. The design space of these 3 analytes was defined by the range in concentration observed in good physical scale‐down models, operated at 10 L scale, of cGMP production bioreactors; 0–10, 0–15, and 0–0.3 g·L^−1^, respectively. A library of historical cell culture supernatant samples was compiled (957 samples) covering this design space for the 3 analytes. The library consisted of historical cell culture supernatant samples taken from a range of fed‐batch cultures using different GS‐CHO cell lines (Lonza Biologics, UK). It included samples from approximately 18 different cell lines grown under many different medium and feed conditions to span as much of the design space as possible. Metabolite data from the library were compiled based on historical values determined using a Nova BioProfile 400 (Nova Biomedical). Design Expert^®^ was used to select samples from the library using the historical metabolite information. An IV‐optimal algorithm was used such that the design space was best represented given the number of samples to be tested (discussed later). Twenty cell culture supernatant samples were selected from the library to assess each spectroscopy technology.

### Spectroscopy techniques

#### FT‐NIR Spectroscopy

A plastic, disposable 96‐well plate (Greiner Bio‐One) was used to hold 20 µL of cell culture supernatant. Each sample was tested in triplicate. Fourier‐Transform NIR (FT‐NIR) spectra were recorded using optic fibers connected to an ABB Bomem FT‐NIR spectrophotometer (MB series). Transmission sampling was carried out by positioning the emitting optic fiber below the plate and collecting by another fiber above the plate using an experimental setup. Spectra acquisition was performed in the range 4000–10,000 cm^−1^ with a 4 cm^−1^ resolution. Sixty‐four scans were taken, resulting in each well taking a total of 1 min 29 s to measure. A background spectrum was measured at the start of each plate by collecting the spectrum of an empty well. The plate was manually positioned to ensure the optic fibers were centered below and above each well at the time of measurement.

#### Raman Spectroscopy

A plastic, disposable 96‐well plate (Nunc™) was used. Automated plate positioning was carried out. Raman spectra were collected with a 785 nm excitation laser using the Raman WorkStation™ microscope (Kaiser Optical Systems). The laser power was 400 mW at the source resulting in ∼100 mW at the sample. An objective with 10× magnification was used and focused with a standard of 20 µL ethanol. Twenty microliters of cell culture supernatant was then analyzed in triplicate. An exposure time of 10 s by 2 scans was used and co‐added to give a total exposure time of 20 s per spectral sample acquisition. Cosmic ray interference removal was applied,^19^, ^20^ resulting in a total processing time of 40 s per sample. Spectra were collected at 0.3 cm^−1^ resolution between 0 and 1900 cm^−1^.

#### 2D‐Fluorescence Spectroscopy

A plastic, disposable 96‐well plate (Greiner Bio‐One) was used to hold 20 µL of cell culture supernatant. Each sample was tested in triplicate. Excitation‐emission matrices (EEMs) were recorded using an at‐line multichannel fluorescence detection system (BioView, Delta Light and Optics, Denmark). Excitation filters ranging from 270 to 550 nm and emission filters ranging from 310 to 590 nm were used, with a filter step‐size of 20 nm for both directions. The plate was manually positioned to ensure the probe was placed directly above the sample.

### Model development

MVDA techniques were used to develop predictive models for glucose, lactate, and ammonium concentration in cell culture supernatant. Spectra of all 3 sample replicates were used for model development (*N* = 60). PLS was applied to develop predictive models using the spectra collected by the FT‐NIR and Raman spectrophotometers. EEMs have increased modes as they incorporate excitation wavelength, emission wavelength, and fluorescence intensity,^21^ resulting in a 3‐dimentional (3D) dataset structure. The analysis of this type of data structure is more complex than that obtained from Raman or NIR spectroscopy. Similar chemometric techniques can, however, be used to decompose the 3D multivariate data such as unfolded‐PLS, NPLS, and PARAFAC (discussed later). Historical metabolite data generated using the Nova BioProfile 400 were used for the calibration of all models developed. The imprecision resolution for each analyte was 5% as reported by the vendor over a range of 0.2–15.0 g·L^−1^, 0.2–5.0 g·L^−1^, and 0.2–25.0 mmol.L^−1^ for glucose, lactate, and ammonium, respectively.^22^ Internal cross‐validation was applied to determine a level of model robustness. Owing to the small sample size, a “Venetian blinds” approach (Eigenvector Research Inc, 2013) was used. Model performance was determined by calculating the root mean square error (RMSE) of calibration (RMSE_C_) and cross‐validation (RMSE_CV_). These performance indicators are used routinely to determine the difference between model predictions and measurement values (Eq. 1)

$$RMSE=\;\sqrt{\frac{\sum_{i=1}^{n}{\left(y_{ref,\;i}-\;y_{model,i}\right)}^{2}}{n}}$$

where y_model__*,i*_ are the values predicted by the model (RMSE_C_) or cross‐validation resampling strategy (RMSE_CV_), and *y*_ref__*,i*_ are the values obtained from the reference method. Outliers were identified by high Hotelling's *T* ^2^ and *Q* residual values (data not shown). The PLS_Toolbox (Eigenvector Research, Inc.) and the N‐way toolbox in Matlab^24^ were used for analysis and model development.

## Results and Discussion

### Design of experiments

Biopharmaceutical process development is moving toward working under a QbD paradigm. QbD involves many steps as defined in the literature,^25^ but one primary difference to traditional manufacturing is defining a process design space. A design space can be defined as “the multidimensional combination and interaction of input variables (e.g., material attributes) and process parameters that have been demonstrated to provide assurance of quality.”^26^ Working within a design space allows greater flexibility but requires a greater understanding of the process. It is extremely important for models to be calibrated using samples that are representative of this design space, to ensure model robustness to variability usually experienced by the process. The investigation presented in this article defined the design space (i.e., the range of concentration) of the 3 analytes according to ranges in concentrations observed in good physical scale‐down models, operated at 10 L scale, of cGMP production bioreactors. Obtaining nonartificial samples spanning the design space is time‐consuming and costly. MB systems have the ability to conduct a large number of controlled, parallel experiments while monitoring several process variables,^27^ which is useful for testing many conditions; however, the same operational constraints are faced with respect to removing samples. Bioanalysers routinely used require a large sample volume, limiting the number of offline measurements that can be taken. Obtaining reference data required for model development is therefore extremely challenging. The methodology presented here utilized samples generated from historical experiments to reduce the time and complexity of obtaining representative samples and enabled reference data already generated to be used for model development.

The literature demonstrates that MB cultures are comparable to those at larger scale.^16^ As a result, it was not important to use samples taken from MB cultures. A library of historical samples for which historical metabolite data was available, was collated that incorporated cell culture supernatant sampled from a range of scales, primarily 10 L cultivations. Spectra of these samples were then acquired under the constraints required for daily monitoring of an MB system.

**Figure 1:**
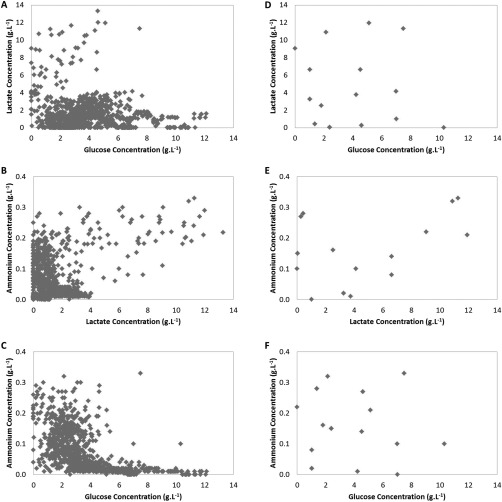
Design space of sample library: glucose (A), lactate (B), and ammonium (C) concentrations of the entire sample library (*N* = 957). (D), (E), and (F) show the subset of the library selected using the DOE approach (*N* = 20).

Figures 1A–C show the analyte concentration distribution of the full library (957 samples) in the selected design space. It can be seen that the samples did not cover the entire design space. In particular, a large area where both ammonium and glucose concentrations are high is not well represented. Subsequent models developed will therefore not capture this behavior and potentially will not be able to accurately predict concentrations within this area of the design space. The library demonstrates, however, that these concentrations are unlikely to be observed during the standard growth cycle of CHO cells, and the omission could therefore be considered physiologically irrelevant from a bioprocessing perspective. As a result, this is not considered a true limitation of the design space, but it might nevertheless limit the capabilities of the MVDA models.

**Figure 2:**
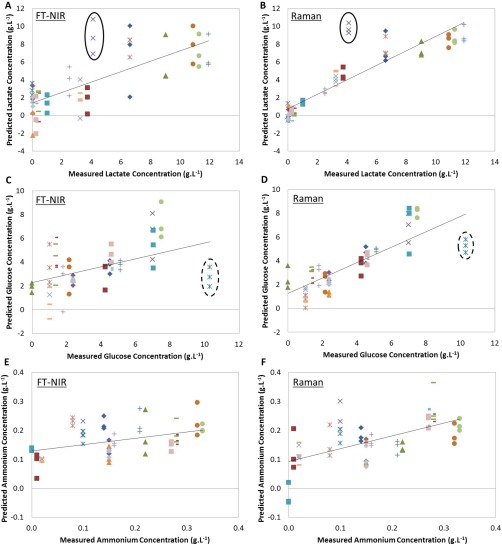
Measured versus predicted plots for individual PLS models: (A) lactate by NIR, (B) lactate by Raman, (C) glucose by NIR, (D) glucose by Raman, (E) ammonium by NIR, (F) ammonium by Raman. Markers indicate replicate measurements from one sample; solid ellipse identifies sample **1** and dotted ellipse sample **2** (*N* = 60 for Raman models and *N* = 59 for NIR models). Solid black line: line of best fit (R2_CV_).

MVDA techniques, such as PLS, were used for model development. These models attempt to describe the largest sources of variation in the dataset. The source of the largest variations within the data set is determined by both the particular form of variation exhibited and the number of samples exhibiting that form of variation. Figures 1A–C display data typical for fed‐batch bioprocesses with the vast majority of the behavior captured by the data being concentrated in a very small part of the design space. This will be reflected in subsequent MVDA models. Samples in unusual outlying parts of the design space are likely to be poorly described by the model (and will appear as outliers having high residuals). For this reason, the authors chose to select a much smaller subset representative of the design space rather than samples representative of the sample library, to develop a robust model able to cope with all the conditions likely to arise. A DOE approach was therefore used to select a subset representative of the design space from the library of historical samples. There are a number of characteristics that should be considered to generate a good experimental design, including, cost effectiveness (i.e., does not require too many runs/samples) and providing a good distribution of the variance of the predicted response throughout the design region.^28^ Ideally, the design should have a low and flat/homologous prediction variance across the design space.^28^, ^29^ Samples were therefore selected from the library by point exchange to satisfy an IV optimality criterion. An IV‐optimal design calculates and minimizes the average or integrated prediction variance over the design space^30^ (Equation 2):

$$IV=\;\frac{1}{A}\int_{R}^{}V\left[y^{pred}\left(x\right)\right]dx$$

where *R* is the design region, *A* is the volume of the region, *V* is the variance, ***x*** are the candidate points, and *y* ^pred^ is the prediction response. A subset of samples representative of the design space was selected from the original library (Figures 2D–F). A subset of 20 samples was selected from the library to enable a fast, efficient approach for comparing the three technologies, which were not all high throughput in the available format.

### Model development

One of the main challenges of using IR to measure analytes in cell culture is its sensitivity to water. NIR spectra obtained primarily represented the first overtone of the O—H stretch in water with a single peak at ∼1400–1500 nm. Its high signal can mask other information in the spectrum. Removing this water peak decreased the model RMSE_CV_ for the lactate concentration model from 2.76 g·L^−1^ to 2.33 g·L^−1^ and increased the variance captured by the model from 78.46% to 86.80%. Water peak removal improved model performance for all other models developed using NIR spectra. Other preprocessing techniques, for example, baseline correction and standard normal variate^20^ that resulted in the best‐performing predictive models, were applied after water peak removal. The raw spectra and one example of preprocessed spectra used for model development are provided in the Supporting Information.

Raman spectroscopy is less sensitive to water interference, and this is advantageous when measuring analytes in cell cultures. However, fluorescence interference can occur as fluorescence has a much stronger signal then Raman scattering,^31^ causing the spectra baseline to shift.^32^ This can be reduced by increasing the wavelength of the laser source^33^ but interference can still occur. Derivative filters and polynomial background fitting can be applied to correct for fluorescence.^32^ Preprocessing methods were applied that resulted in the best‐performing predictive models. Raw Raman spectra and one example of preprocessed spectra used for model development are provided in the Supporting Information.

Fluorescence EEMs also require preprocessing, as both Raman and Rayleigh scattering can cause interference and bias the estimated model parameters.^34^ This is particularly important when using PARAFAC to analyse the data. PARAFAC decomposes the fluorescence signal into a number of trilinear structures. Rayleigh and Raman peaks do not follow trilinearly, as, for example, the shape and position of the peaks changes with excitation wavelength.^35^ Discarding these peaks and replacing with zeros can remove this signal interference while still maintaining some chemical and physical information.^36^ This method was applied prior to model development.

#### NIR and Raman Predictive Models

Individual PLS models were developed to predict lactate, glucose, and ammonium concentration in cell culture supernatant samples using the preprocessed NIR spectra and Raman spectra. Models developed from Raman spectra used the fingerprint region of the spectrum (400–1800 cm^−1^). The optimal number of latent variables (LVs) to include in each model was determined by comparing RMSE_CV_ and RMSE_C_ values to prevent overfitting.^23^ Figure 2 displays the measured versus predicted plots of the PLS models developed for lactate concentration in cell culture supernatant using NIR spectra (A) and Raman spectra (B), glucose concentration using the NIR spectra (C) and Raman spectra (D), and ammonium concentration using the NIR spectra (E) and Raman spectra (F).

**Figure 3:**
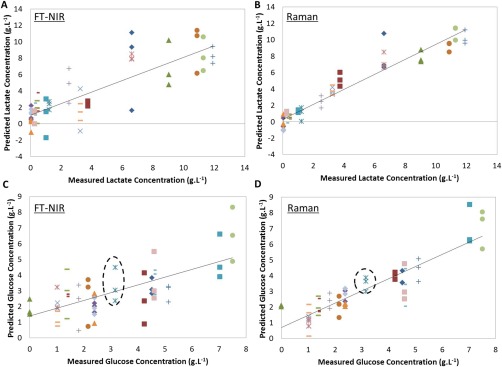
Measured versus predicted plots for the final PLS models developed: (A) lactate by NIR, (B) lactate by Raman, (C) glucose by NIR, and **(**D) glucose by Raman. Dotted ellipse identify sample **2** (*N* = 57 for Raman models, *N* = 56 for NIR models). Solid black line: line of best fit (R2_CV_).

The investigation identified 2 cell culture supernatant samples whose values were inaccurately predicted by the models developed using the NIR and Raman technologies (samples denoted **1** and **2**). Both NIR (Figure 2A) and Raman (Figure 2B) predict the lactate concentration in sample **1** (ellipse) to be ∼9–10 g·L^−1^ instead of ∼4 g·L^−1^ as measured by the reference method (Nova BioProfile 400). The glucose and ammonium measurements of this sample were, however, accurately predicted (purple crosses in Figures 2C–F). Similarly, both technologies were unable to accurately estimate glucose concentration in sample **2**, predicting the value to be ∼3 g·L^−1^ instead of 10 g·L^−1^ (identified in Figures 2C and D). To evaluate the capabilities of both technologies further, the accuracy and precision of the predictions for these two deviating samples was examined (Table 1). The RMSE_CV_ was used as a measure of accuracy of the model to predict each sample. The median absolute deviation (MAD) was calculated as a measure of the precision^37^ (Equation 3)

$$MAD=\;Median\left(\left|Y_{i}-Y∼\right|\right)$$

where *Y*∼ is the median of the data and |*Y*| is the absolute value. Despite relatively high RMSE_CV_ values, both the NIR and Raman predicted similar concentrations for the analytes and with relatively good precision. Owing to the close agreement between technologies with regards to the predicted metabolite concentration and the lack of error in the models developed to measure ammonium concentration, it was hypothesized that errors exist within the historical metabolite concentration data for these two samples. Concentrations of the 3 analytes were therefore redetermined using the reference method. New measurements of sample **2** identified errors with the historical data; historical lactate concentration was 0 g·L^−1^, whereas the new reading measured 1.21 g·L^−1^; and the historical glucose concentration was 10.31 g·L^−1^, whereas the new reading measured 3.16 g L^−1^. This repeated reference analysis could not be carried out for sample **1** due to insufficient volume available. New models were developed using the new measurement of sample **2** and removing the concentrations determined for sample **1** from the dataset (Figure 3). This improved the model performance as determined by RMSE_CV_ values.

**Table 1:** Precision and Accuracy of the Raman and NIR Predicted Values for Lactate and Glucose Concentration in Sample 1 and 2.

Sample	Technology	Analyte	Figure	Precision (g·L^−1^)	Accuracy (g·L^−1^)
1	NIR	Lactate	2A	1.78	4.92
1	Raman	Lactate	2B	0.36	5.63
2	NIR	Glucose	2C	0.78	7.59
2	Raman	Glucose	2D	0.52	5.09

For indirect statistical models such as PLS, the error of each model is a combination of the error in the reference method and the spectroscopy analysis signal. The methodology presented in this article utilized historical samples to reduce the time and complexity of obtaining non‐artificial samples spanning the process design space. Confidence in the accuracy of the historical data is therefore important to the overall error of the models developed. This investigation highlights the importance of reference method accuracy and having confidence in historical data. In addition, both NIR and Raman spectroscopy technologies were able to identify these outliers in the historical data despite model calibration incorporating these errors. This further highlights the ability of both of these technologies to measure each of the analytes. The new values were used for model development with 2D‐F EEMs.

#### 2D‐Fluorescence Predictive Models

2D‐F produces a 3D dataset by stacking EEMs from different samples. There are a number of different methods to analyze EEMs. One method routinely used to analyze fluorescence data involves unfolding the 3D‐dataset as described by Nomikos and MacGregor (1994) for batch process data.^17^ The dataset is unfolded from an *I* × *J* × *K* cube into a new matrix of size *I* × *JK* so standard PLS can be used with the new **X** dataset. There are a number of examples where an unfolding approach has been carried out on 2D‐F EEMs,^38^, ^39^, ^40^ but it has been argued that unfolding the dataset loses additional information gained from the 3D structure. Other analysis techniques referred to as multiway analyses have been applied to fluorescence data, specifically NPLS^21^ and PARAFAC. NPLS is very similar to PLS; however, the multidimensional structure is maintained by decomposing the multiway **X** into, for example, a set of triads consisting of one score vector and two weight vectors. PARAFAC is a trilinear unsupervised decomposition method often used for exploratory analysis. It decomposes the dataset into trilinear components, resulting in two loading matrices and one scores or concentration‐related matrix. PARAFAC is not a regression method; however, it has been shown that PLS can be carried out on PARAFAC scores to develop predictive models.^41^ All 3 analysis techniques were compared in this investigation.

**Figure 4:**
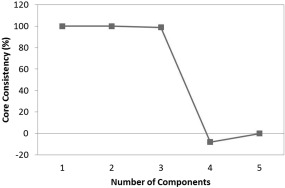
Core consistency calculated for 5 individual PARAFAC models of increasing model complexity.

Determination of the number of components to include in the NPLS and PLS models was carried out as per the NIR and Raman spectra. Different methods were used for the PARAFAC models: core consistency and split‐half analysis.^42^ For the core consistency diagnostic method, 5 individual PARAFAC models were developed, containing from 1 to 5 components using the N‐way toolbox in Matlab.^24^ The core consistency shows the difference between the core array of a PARAFAC model compared to the “ideal.” If the core array is equal to the “ideal,” then the score is 100%. The results demonstrate that 3 components should be included (Figure 4). However, core consistency is not always a conclusive procedure. Split‐half analysis was also performed. This method involves splitting the dataset into 2 (or more) sections and developing individual PARAFAC models on each. If the correct number of components has been included in the model then, due to the uniqueness property of the PARAFAC decomposition, the same loadings will be found for each separate model built on the subsections.^43^ This test was performed on the EEMs, where the data were split into 2 subsections, avoiding blocking issues due to the repeated measurements. Two PARAFAC models were developed on each subsection, 3 and 4 component models. Unlike the core consistency analysis, no difference was observed between subsections for the 3 component model (99.9% similarity) or the 4 component model (98.9% similarity). Two PLS models were therefore evaluated; one using scores from the 3 and one from the 4 component PARAFAC models (Table 2).

**Table 2:** Comparison of Models Developed with the Fluorescence EEM Using Three Different Model Building Techniques: Unfolded‐PLS, NPLS, and PARAFAC‐PLS.

Model building method	Latent variables	RMSE_C_ (g·L^−1^)	RMSE_CV_ (g·L^−1^)	$R_{\text{C}}^{2}$	R2_CV_
**Lactate concentration**					
Unfolded PLS	9	0.65	1.06	0.975	0.935
NPLS	6	1.81	2.01	0.808	0.770
PARAFAC 3 component/PLS	3	2.82	2.91	0.533	0.502
PARAFAC 4 component/PLS	4	2.80	2.95	0.538	0.490
**Glucose concentration**					
Unfolded PLS	4	1.36	1.53	0.560	0.442
NPLS	5	1.34	1.43	0.572	0.513
PARAFAC 3 component/PLS	2	1.86	1.91	0.166	0.130
PARAFAC 4 components/PLS	4	1.80	1.92	0.220	0.133
**Ammonium concentration**					
Unfolded PLS	9	0.021	0.031	0.960	0.917
NPLS	6	0.041	0.054	0.850	0.748
PARAFAC 3 component/PLS	3	0.084	0.086	0.367	0.342
PARAFAC 4 components/PLS	3	0.082	0.084	0.391	0.363

**Figure 5:**
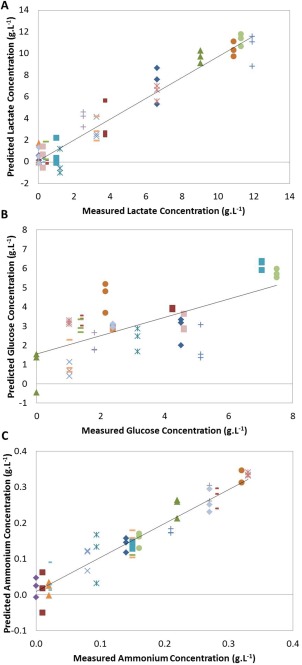
Measured versus predicted plots for the unfolded PLS models developed using 2D‐fluorescence EEMs: (A) lactate, (B) glucose, and (C) ammonium concentrations (*N* = 56). Solid black line: line of best fit (R2_CV_).

Unfolding‐PLS and the NPLS methods developed more accurate models for estimating the concentrations of the 3 analytes than applying PLS on PARAFAC scores. These findings agree with those of Mortensen and Bro (2006). PARAFAC is an unsupervised decomposition method that provides the most chemically relevant information but not necessarily the most optimal solutions for predictions.^41^ PLS and NPLS are calibration methods that result in a least‐squares solution to obtain maximal covariance between **X** and **Y** 18 explaining their ability to develop more accurate predictive models than the unsupervised PARAFAC method. Overall, the unfolding‐PLS method produced better or similar results in predicting lactate, glucose, and ammonium concentration in 20 µL cell culture supernatant (RMSE_CV_: 1.06, 1.53, and 0.031 g·L^−1^, respectively) (Figures 5A–C). This suggests that maintaining the 3D‐structure of the dataset is not necessary to obtain sufficient information for predicting the 3 analytes in cell culture supernatant. It is interesting to note that the models developed using unfolded‐PLS required more LVs compared to NPLS to model the behavior of the samples. This could be a result of losing the overall 3D‐structure.

### Comparison of technologies

The aim of this study was to determine which of the 3 spectroscopy technologies could more accurately measure the concentration of 3 analytes under set criteria, small sample volume and minimal acquisition times.

For routine operation of the NIR and 2D‐F spectrophotometers, a dedicated plate reader would need to be attached to allow for automated spectra acquisition in each well. Further studies are therefore required to test any potential reduction in sensitivity resulting from the application of the automated technology. The optimized predictive models of each analyte developed in this investigation were however compared. The RMSE_C/CV_ and *R* ^2^ coefficient were calculated for each model using an internal cross‐validation approach as carried out in the literature.^4^, ^44^ In addition, precision was also calculated. These were used to assess the performance of each model and compare the technologies (Table 3). Raman and 2D‐F could measure lactate concentration to within a similar accuracy (RMSE_CV_ 1.11 g·L^−1^ and 1.06 g·L^−1^, respectively) and outperformed NIR (RMSE_CV_ 2.33 g·L^−1^). The lactate model developed using the Raman spectra, however, demonstrated better precision compared to that using the fluorescence EEMs (MAD 0.29 g·L^−1^ and 0.43 g·L^−1^, respectively). Raman spectroscopy was considerably more accurate at measuring glucose concentration (RMSE_CV_ 0.92 g·L^−1^) compared to both 2D‐F and NIR (RMSE_CV_ 1.53 g·L^−1^ and 1.50 g·L^−1^, respectively) and still demonstrated a good level of precision (0.31 g·L^−1^). NIR demonstrated good precision (0.012 g·L^−1^) for predicting ammonium concentration; however, the model had a much higher RMSE_CV_ and the *R* ^2^ coefficient demonstrated the predictions had a much weaker linear correlation to the measured values ( $R_{\text{CV}}^{2}$ 0.218). 2D‐F achieved a higher accuracy than both the other methods for measuring ammonium concentration with an RMSE_CV_ 0.031 g·L^−1^ compared to RMSE_CV_ values of 0.072 g·L^−1^ and 0.093 g·L^−1^ demonstrated by the models developed using Raman and NIR spectra, respectively.

**Table 3:** Comparison of the Three Different Spectroscopy Techniques for Measuring Lactate, Glucose, and Ammonium Concentration in Cell Culture Supernatant.

Technology	Model building method	Latent variables	RMSE_C_ (g·L^−1^)	RMSE_CV_ (g·L^−1^)	$R_{\text{C}}^{2}$	$R_{\text{CV}}^{2}$	Precision (g·L^−1^)
**Lactate concentration**							
2D‐Fluorescence	Unfolded PLS	9	0.65	1.06	0.975	0.935	0.43
Raman	PLS	6	0.86	1.11	0.957	0.927	0.29
NIR	PLS	9	1.51	2.33	0.868	0.686	0.79
**Glucose concentration**							
2D‐Fluorescence	Unfolded PLS	4	1.36	1.53	0.560	0.442	0.17
Raman	PLS	8	0.46	0.92	0.949	0.798	0.31
NIR	PLS	7	1.09	1.53	0.717	0.452	0.51
**Ammonium concentration**							
2D‐Fluorescence	Unfolded PLS	9	0.021	0.031	0.960	0.917	0.015
Raman	PLS	5	0.064	0.072	0.636	0.533	0.023
NIR	PLS	3	0.087	0.093	0.304	0.218	0.012

Comparison of the performance indicators of the PLS models developed suggest that Raman or 2D‐F would be the most suitable technology for measuring glucose, lactate, and ammonium under operational conditions suitable for daily monitoring of a MB system. The intensity of Raman scattering is dependent on numerous factors, including the polarizability of the molecule and the concentration of the analyte. The models developed using Raman spectra may have a higher error for measuring ammonium concentration compared to 2D‐F for this specific application as a result of the low concentrations being measured and the low polarizability of the molecule compared to glucose and lactate (glucose: 15.16 Å^3^, lactate: 7.43 Å^3^, ammonia: 2.26 Å^3^).^45^ 2D‐F has a greater sensitivity than both Raman and NIR spectroscopy,^46^ making the technology more adapt to measuring low analyte concentrations. 2D‐F can, however, only detect molecules with intrinsic fluorescence properties. The ability of a molecule to fluoresce is related to the extent of conjugation within the molecule as a result of weaker π‐bonds. The less strongly bound electrons can be excited by photons with lower energy.^47^ As a result, aromatic and unsaturated compounds are more likely to absorb in the UV–vis region. Despite this, a number of examples in the literature demonstrate the ability to develop PLS models using 2D‐F EEMs to predict the concentration of glucose, lactate, and ammonium.^9^, ^10^ These models, however, rely on correlations with other factors such as fluorophores involved in cell growth and glucose consumption.^10^ Models developed on indirect measurements are susceptible to metabolic and process parameter changes.^7^ As a result, models developed using 2D‐F EEMs for this application are highly dependent on the matrix background and cell lines and may demonstrate little robustness to process changes.

MVDA was used in this investigation for developing individual predictive models measuring lactate, glucose, and ammonium concentration. Decomposition methods such as PLS reduce the number of variables to a smaller set of uncorrelated LVs that explain co‐variance with the reference values and the variance in the spectra. Highly complex models were developed for the majority of analytes as a result of the large number of LVs included. The requirement for a large number of LVs may have been a result of our calibration approach, which deliberately broke the correlations between the variables.

Overall, the model errors reported in this investigation are higher than those published in the literature. However, the literature primarily demonstrates examples where spectroscopy technologies are implemented online and whose applications can allow longer acquisition times, for example, 600 s.^48^ The application presented in this investigation requires the results of all 48 MBs to be obtained in 1 h. As such, this application is very challenging. Small acquisition times reduce the signal‐to‐noise ratio of spectra and will therefore affect the performance of subsequent models developed.

## Conclusion

A novel approach using DOE methodology was described that utilized historical cell culture supernatant. The use of historical samples reduced the time and complexity of an experiment required to obtain a nonartificial diverse sample set that also spanned the relevant design space. This study did, however, demonstrate the importance of having confidence in the historical measurements to develop accurate predictive models. Further work is required to improve the predictive capability of the selected technology for accurate monitoring and control of the process. However, this methodology enabled a quick and effective approach for the technologies to be compared with samples representative of the design space. In addition, operational constraints specific to MB systems were established, in particular the small volume size and short acquisition times. The experimental comparison demonstrated that either 2D‐F or Raman spectroscopy could more accurately measure lactate, glucose, and ammonium concentration in cell culture supernatant for this application, than NIR. The data analysis for the 2D‐F EEMs was, however, much more involved than the Raman spectra and the final models may not be as robust to process changes due to the reliance on correlations with other components. This study therefore identified Raman spectroscopy to be the most suitable technology, at present, for this application. The implementation of Raman spectroscopy would enable daily monitoring of key cell culture components, increasing potential applications for MB systems.

## Supporting information


**S1.** Displays the raw spectra acquired using NIR spectroscopy (A) and Raman spectroscopy (C). Examples of pre‐processing methods applied to the raw spectra are also shown; standard normal variance followed by a Savitzy‐Golay smoothing filter (15 filter width) was applied to the NIR spectra after water peak removal (B) and to the Raman spectra (D).
