# Supplementary material for: Comparison of spectroscopy technologies for improved monitoring of cell culture processes in miniature bioreactors
Source: Biotechnol Prog. 2017 Mar 29;33(2):337–46. doi: 10.1002/btpr.2459 (PMC5413828; doi:10.1002/btpr.2459)
Supplement: Supplementary file 1 — S1. Displays the raw spectra acquired using NIR spectroscopy (A) and Raman spectroscopy (C). Examples of pre‐processing methods applied to the raw spectra are also shown; standard normal variance followed by a Savitzy‐Golay smoothing filter (15 filter width) was applied to the NIR spectra after water peak removal (B) and to the Raman spectra (D). [file BTPR-33-337-s001.docx]

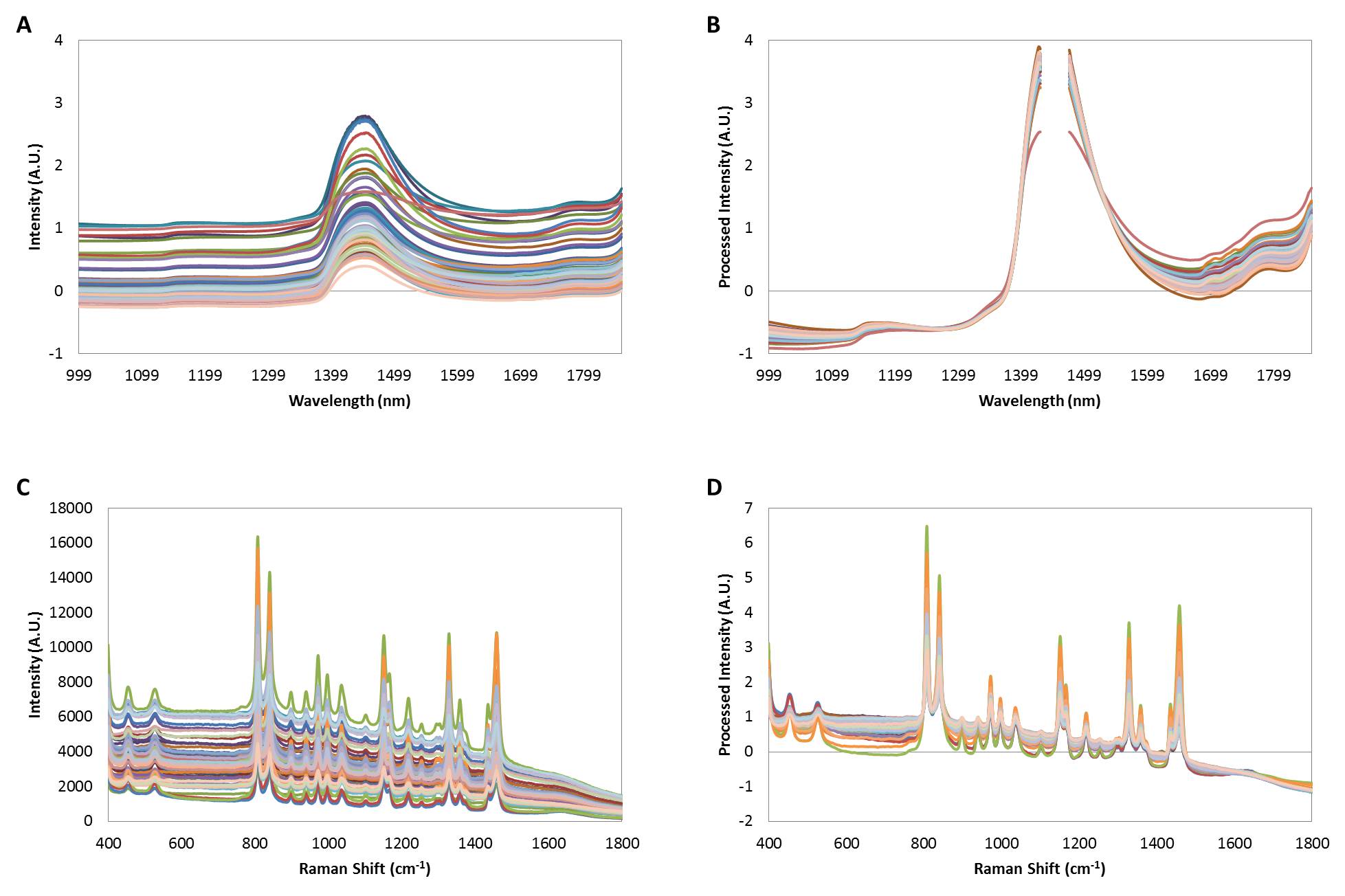


S1. Displays the raw spectra acquired using NIR spectroscopy (A) and Raman spectroscopy (C). Examples of pre-processing methods applied to the raw spectra are also shown; standard normal variance followed by a Savitzy-Golay smoothing filter (15 filter width) was applied to the NIR spectra after water peak removal (B) and to the Raman spectra (D).
